# Glucagon Stimulation Testing in Assessing for Adult Growth Hormone Deficiency: Current Status and Future Perspectives

**DOI:** 10.5402/2011/608056

**Published:** 2011-08-11

**Authors:** Kevin C. J. Yuen

**Affiliations:** Division of Endocrinology, Diabetes and Clinical Nutrition, Oregon Health and Science University, Portland, OR 97239-3098, USA

## Abstract

Growth hormone deficiency (GHD) is a well-recognized clinical syndrome in adults. However, due to the high frequency of normal serum IGF-I levels in hypopituitary adults with GHD, it is now widely accepted that despite normal levels of total IGF-I, adults clinically suspected with GHD within the appropriate clinical setting must undergo GH provocative testing to confirm its diagnosis. Although the insulin tolerance test (ITT) is labor intensive, contraindicated in the elderly and in adults with seizure disorders and ischemic heart disease, can be unpleasant for the patient, and is potentially hazardous, this test remains the gold standard test for the biochemical demonstration of GHD in adults. In contrast, with the unavailability of the GHRH and arginine test as the alternative test to the ITT in the United States since 2008, the glucagon stimulation test (GST) has since been increasingly used in the United States because of its availability, reproducibility, safety, lack of influence by gender and hypothalamic cause of GHD, and relatively few contraindications. In this paper, we discuss our recommendations in performing this test, the potential drawbacks in conducting and caveats in interpreting this test, and its future perspectives.

## 1. Introduction

Growth hormone deficiency (GHD) in adults is characterized by alterations in body composition, carbohydrate and lipid metabolism, bone mineral density, cardiovascular risk profile, and quality of life [1]. Treatment with GH replacement has been shown to improve some, but not all, of these abnormalities [2]. In contrast, untreated GHD is associated with increased mortality and morbidity that was previously observed in adults with hypopituitarism [3, 4]. These findings were substantiated in two large surveys based on national Danish registries [5, 6]. In the study by Stochholm et al., the authors reported that overall mortality was increased in adults with GHD, with female patients having significantly higher mortality rates compared to males, and patients with childhood onset far exceeding that of adult-onset GH deficient patients [5]. In another study published a year later, the same authors reported that the morbidity of adults with GHD was approximately threefold higher than that of a healthy population, with implicated causes being due to infectious, cancer, endocrine, neurological, eye, ear, circulatory, pulmonary, and urogenital diseases, and trauma [6]. This result was independent of gender and applied to patients with childhood-onset and adult-onset GHD. 

Current published guidelines recommend the evaluation of adult GHD to be based on clinical findings, medical history, and using the appropriate GH stimulation test for biochemical confirmation [7–9], with the exception of patients with three or more pituitary hormone deficiencies and low serum IGF-I levels [10]. Serum IGF-I levels should not be used alone to diagnose adult GHD, and the maximum or peak GH secretion following GH stimulation testing is used as a surrogate of the capacity of the pituitary to release GH. The insulin tolerance test (ITT) is generally considered the gold standard test for the evaluation of GH deficiency and has been endorsed by several consensus guidelines [7–9, 11]. However, this test is labor intensive, may be unpleasant for some patients, has potential risks, and is contraindicated in elderly patients and in patients with seizure disorders and ischemic heart disease. Thus, there remains a real unmet medical need for an alternative test to the ITT that is safe yet reliable. For this reason, several other dynamic tests have been proposed such as arginine (ARG), combined GH releasing hormone plus ARG (GHRH-ARG), and levodopa (L-DOPA) in spite of data indicating poor performance of some of these tests for evaluation of adult GHD [10, 12]. A potential alternative to the ITT is the glucagon stimulation test (GST) that has been used extensively in the United Kingdom [13] and is gradually gaining acceptance in the United States [14]. 

## 2. Update on the Glucagon Stimulation Test in Diagnosing Adult GHD

Following the publication of several validation studies [12, 15–17] and recommendations from current consensus guidelines [7–9, 11], the GHRH-ARG test has in recent years emerged as the best and most reliable alternative GH stimulation test to the ITT in diagnosing adult GHD. However, when EMD Serono, Inc decided to discontinue the manufacture of recombinant GHRH (Geref) in the United States in July 2008 [18], this inevitably left a significant gap for an alternative reliable test for the evaluation of patients suspected to have GHD in place of the GHRH-ARG test. This is particularly important for endocrinologists in the United States who are not comfortable or do not have the necessary logistic or staff support to conduct ITTs in their office or patients who have contraindications to hypoglycemia in whom the ITT would be inappropriate. With the lack of reliable GH stimulation tests available in the United States, we have recommended the glucagon stimulation test (GST) as the alternative test to the ITT for diagnosing adult GHD based on its availability, reproducibility, safety, lack of influence by gender and hypothalamic cause of GHD, and relatively few contraindications [14]. 

Analyzing the data of 13,167 GH-deficient patients enrolled in the KIMS pharmacoepidemiological database (Pfizer International Metabolic Database) from its inception to the end of 2008, Brabant et al. addressed the question of whether there were regional differences in the use of different biochemical tests to diagnose adult GHD in 6 large European countries and the United States [13]. This analysis revealed striking regional variations in the approach to GH stimulation testing. The ITT was found to be the most popular test used in 44.3% of all countries but was less popular (13.3%) in the United States ranking second after the arginine test, whereas the GST was ranked third in the United States, being the most popular in the United Kingdom (29.9%) and the least popular in Germany and the Netherlands (0.1%). However, the unavailability of the GHRH-ARG test in the United States since 2008 has resulted in a change in recent years with the GST being more frequently used as the alternative test to the ITT [14]. 

The use of the GST for the assessment of GH reserve was first described in 1969 by Mitchell et al. [19]. Since then, the GST has been shown by various investigators to have a GH secretory potency that is similar to or only slightly less than the ITT, suggesting that it is more reliable than other classic agents such as ARG or clonidine for separating GHD patients from normal subjects [20–24]. The true mechanism by which glucagon induces GH release remains unclear. Some of the hypothesized mechanisms include the glycemic fluctuations during the test where blood glucose levels increase initially before decreasing later in the test [25], the generation of a peptidyl fragment associated with the GH- and ACTH-releasing activity [26] and the induction of norepinephrine secretion in stimulating GH release via *α*-receptors [27]. It has also been previously demonstrated that glucagon stimulates GH release more effectively when administered intramuscularly or subcutaneously as opposed to the intravenous route [23]. 

The three studies utilizing the GST by Gomez et al. [28], Conceicao et al. [22], and Berg et al. [21] evaluated GHD in patients with pituitary disorders. The first two studies [22, 28] were prospective studies that compared the diagnostic characteristics of GST to ITT and included a control group which was matched for age and sex in both studies and for body mass index (BMI) in one study [28]. Using receiver-operator curve (ROC) analysis, both studies proposed a peak GH cutoff value of 3 ng/mL as the best cutpoint with the highest combined sensitivity and specificity to differentiate between patients with GHD and healthy controls [28, 29]. Additionally, Gomez et al. [28] found no correlation among age, sex, and BMI with peak GH levels in patients with hypopituitarism, but there was a significant negative correlation between age (*r* = −0.389, *P* = 0.0075) and BMI (*r* = −0.329, *P* = 0.025) with peak GH levels in healthy controls. It is important to note that the GH-deficient adults in this study had higher BMIs than the healthy controls; nevertheless, these data suggest that there is a potential association between relative, but not functional, GH deficiency of obesity and aging with BMI. By contrast, the study by Conceicao et al. [22] demonstrated that peak GH levels were not affected by age in either the control or patient group, and that there were no gender differences. It is, however, noteworthy that in the study by Gomez et al. [28], the dose of glucagon administered was 1 mg for subjects that weighed 90 kg or less and 1.5 mg for subjects that weighed more than 90 kg whereas in the study by Conceicao et al. [22], all subjects received 1 mg of glucagon. Furthermore, there were slightly more females in the study by Conceicao et al. [22] compared to Gomez et al. [28] study. On the other hand, the study by Berg et al. was a retrospective study that revealed an optimal peak GH cutoff value of 2.5 ng/mL with 95% sensitivity and 79% specificity using ROC analysis [21]. This study also reported lower peak GH levels with GST compared to ITT (5.1 versus 6.7 ng/mL, *P* < 0.01) but a significant positive correlation between peak GH levels during ITT and GST (*r* = 0.88, *P* < 0.0001). Additionally, no correlation between BMI and age to peak GH responses was observed, peak GH responses occurred mainly between 120 and 180 min consistent with previous studies [27, 30], and that, overall, the GST was a well-tolerated test. Nevertheless, these [21, 22, 28] and previous studies [20, 23, 24, 31] did not specifically evaluate patients with glucose intolerance and frank diabetes, and for this reason, the characteristic of the GST and its reliability in testing for GHD in this population remains unclear. This is especially important since performing ITT in patients with diabetes is usually challenging and may not be safe especially if large insulin doses are required to achieve hypoglycaemia in patients with underlying insulin resistance. 

## 3. Other Considerations in Performing and Interpreting the Data of the GST

The diagnosis of adult GHD has proved to be challenging because of the lack of a single biological endpoint such as growth failure, and, therefore, the confirmation of adult GHD largely depends on biochemical provocative testing. Clearly, there is no ideal stimulation test and we recommend that the decision to embark on a stimulation test, to diagnose adult GHD must factor in the appropriate clinical context of each individual patient together with the number of pituitary hormone deficiencies plus serum IGF-I level [10], the validity of the chosen test and its appropriate cutoff limits, the sensitivity of the GH assay, and the availability of local resources and expertise. 

The GST is a simple and safe test to perform (Table 1). Glucagon is readily accessible because it is widely available for treating hypoglycemic episodes in patients with diabetes. In addition, glucagon is relatively inexpensive (the current average wholesale price of recombinant DNA glucagon is approximately $50–$70 per single 1 mg dose, while for Geref and ARG is approximately $80–$130 per single 50 *μ*g and $10–$12 per single 30 g dose, resp.). Glucagon appears to be welltolerated and is only relatively contraindicated in patients with malnourishment or patients who have not eaten for more than 48 hours due to concern of prolonged hypoglycemia and those with pheochromocytoma in whom a significant exacerbation of blood pressure may be observed [25]. 

The GST was initially described as a 4-hour test in older studies [32, 33], but more recent studies have suggested that the test could be shortened to a 3-hour test, and that serum GH levels can be evaluated between 3 to 5 time points only (0, 90, 120, 150, and 180 mins) as the majority of GH peaks occurred between 120 and 180 mins (85%) [27, 31]. In a study by Orme et al. comparing standard and simplified GST (0, 150 and 180 min), 75% of discordant GH results were due to a peak GH level occurring at 210 min [31]. Accordingly, the authors proposed that the diagnostic utility of the simplified GST could be improved further by drawing an additional blood sample at 210 min when assessing GH deficiency. The audit by Leong et al. [27] is the largest study that assessed patients with hypothalamic-pituitary disease whom had undergone the GST, and they reported that the test could be shortened by omitting the 240-min blood sample. Among 414 patients who underwent GSTs, the majority of peak GH levels occurred between 120 and 180 min (85%) and 5 patients had their peak GH levels recorded at 240 min. Hence, it is still not clear whether the ideal timing of the GST is 3 versus 4 hours, and continuing the test for 4 hours may be advisable, at least until there are more definitive data available. This also allows the monitoring for late hypoglycemia, although truly low blood glucose levels are not common. While the lowest blood glucose level with the GST in the literature was reported at 37 mg/dL [19], in our experience [34], we rarely observed blood glucose levels falling below 40 mg/dL with this test. The occurrence of hypoglycemia reported in the literature with blood glucose levels lower than 40 mg/dL during GSTs is also rare event [21, 27]. 

The common sideeffects in patients with hypothalamic-pituitary disease that underwent testing with the GST included nausea, vomiting, and headaches and have been reported to range from less than 10 [21] to 34% [27]. In a study of 97 normal subjects, mild nausea in approximately 30% of the subjects and transient vomiting and retching in about 10% of the subjects were the only side effects that were noted [35], whereas in our experience of 143 GSTs performed at 4 large academic centers in the United States, the main side effects reported were nausea (41%), fatigue, headaches, weakness, and hunger (12%) [34]. 

Like other GH stimulation tests, there are also limitations associated with the GST. The 3- or 4-hour GST is still longer than many other GH stimulation tests and requires an intramuscular injection which may not appeal to some patients. However, as there is a relationship between peak GH response to GHRH-ARG stimulation and ambient glucose levels [29], it is unclear whether hyperglycemia may play a part in influencing the peak GH response to glucagon stimulation. Furthermore, no peak GH responses have been studied using the GST in normal controls over the age of 70 years, and none of the previous studies included patients with frank diabetes. Therefore, it is not known whether testing using the GST in subjects with diabetes is valid. Hence, caution should be exercised when interpreting normal GST results in the patients with diabetes. If the suspicion of GHD remains high in these patients, it is reasonable to consider using a second GH stimulation test. Finally, while it is accepted that a peak GH response of 3 ng/mL or less is the best cutpoint to diagnose adult GHD using the GST [22, 28], there remains a lack of consensus over a peak GH response between 3 ng/mL and 10 ng/mL, and further studies are required to address this. 

Other provocative tests that have been proposed include ARG alone and GH secretagogues. ARG alone has been shown to be less reliable than the ITT or GHRH-ARG [12] and the mean peak GH response to ARG alone is lower than in the ITT or GST, even in normal lean subjects [24]. The diagnostic reliability of ARG alone has been previously questioned [12, 20]. Thus, we recommend that ARG alone should only be considered if the ITT and the GST is contraindicated or if glucagon is unavailable. If this test is used, appropriately low peak GH cutoffs should be employed (for 95% sensitivity: 1.4 *μ*g/L, for 95% specificity: 0.21 *μ*g/L, and to minimize misclassification in either direction: 0.4 *μ*g/L) [12]. In contrast, the reliability of testing with GH secretagogues such as GH-releasing peptide-2 alone [36], GH-releasing peptide-6 alone, and combined GH-releasing peptide-6 plus GHRH [37] in comparison with the ITT has also been demonstrated. These agents utilize the same concept as the GHRH-ARG test in stimulating pituitary GH release by mimicking the activity of the natural GH secretagogue receptor ligand (i.e., ghrelin) and appear to demonstrate a good safety profile with relatively few contraindications [38]. The limitation, however, of these GH secretagogues is that these agents are more likely to explore the pituitary somatotroph releasable pool and might potentially induce misleadingly normal peak GH responses in hypothalamic GHD [39]. Furthermore, these agents are not readily available in many countries including the United States.

## 4. Future Perspectives

Recent studies have indicated that further refinements to the GST may still be required to improve the sensitivity and specificity of this test. A study by Micmacher et al. [40] demonstrated in a group of healthy men above 50-year old that GH secretion in response to the GST, but not with the ITT, correlated to physiological spontaneous GH secretion. These data indicate that GH response to the GST reflects the endogenous GH spontaneous secretion and poses the question as to whether the cutpoints of peak GH response to the GST should be agedependent. More recently, we have reported a 1-year experience of GSTs conducted from 4 large academic centers in the United States and explored the potential of the GST in testing the hypothalamic-pituitary-adrenal axis [34]. In this study, we found that the majority of GH peaks occurred between 120 and 180 mins (78%) and that there was a negative correlation between fasting glucose (*r* = −0.24, *P* < 0.01) and BMI (*r* = −0.37, *P* < 0.01) with peak GH levels. When compared with the 250 *μ*g cosyntropin stimulation test using a cutpoint of 18 *μ*g/dL, peak cortisol levels with GSTs were lower (*P* < 0.02) and had higher failure rates (44.4% versus 33.3%), and the 120-min peak cortisol of 16.5 *μ*g/dL achieved 83.3% sensitivity and 75% specificity using ROC analysis. Overall, the GST was well tolerated and can be performed as an outpatient; however, further studies are required to determine whether GSTs may falsely diagnose GHD in patients with fasting hyperglycemia, and/or high BMIs. Thus, to improve the diagnostic reliability of the GST especially in patients with glucose intolerance and in those with high BMIs, a priming agent may be required to combine with the GST with appropriate cutpoints to improve its sensitivity and specificity, similar to the GHRH in priming the ARG test. Until such data becomes available, we recommend that a second GH stimulation test should still be considered for such patients. 

In conclusion, in line with recently published consensus guidelines [7–9, 11], the ITT should remain as the test of reference due to its greatest diagnostic accuracy, even in patients with suspected hypothalamic GHRH deficit. We recommend the GST as the alternative test to the ITT for diagnosing adult GHD because of its availability, reproducibility, safety, lack of influence by gender and hypothalamic cause of GHD, and relatively few contraindications. Despite some studies demonstrating the comparability of the GST to the ITT in assessing the hypothalamic-pituitary-adrenal axis [41, 42], further larger, well-controlled studies are still needed to confirm the reliability of the GST in assessing this axis. If the GST can be shown to reliably distinguish adrenal sufficiency from insufficiency, then the ability of assessing both the GH and cortisol reserve simultaneously, just as in the ITT, would make this test even more appealing. While previous studies have shown that the GST could be shortened from 4 to 3 hours and yet maintain its diagnostic utility [27, 31], until further data becomes available, we would still recommend that the GST be conducted over 4 hours with measurements every 30 min for serum GH and capillary blood glucose levels primarily to ensure that delayed peak GH responses and late hypoglycemia are not missed. 

##  Conflict of Interests

K. C. J. Yuen declares that there is no duality or conflict of interest to disclose.

## Figures and Tables

**Table 1:** Recommended protocol for performing the GST in adults.

*Contraindications:*	
Malnourished patients or patients who have not eaten for >48 h	

*Precautions: *	
Patients may feel nauseous during and after the test (administration of intravenous antiemetics can be considered)	
Late hypoglycaemia may occur (patients should be advised to eat small and frequent meals after the completion of the test)	

*Procedure:*	
Ensure patient is fasted from midnight	
Weigh patient	
Patient in recumbent position and intravenous cannula inserted for intravenous access between 8 am to 9 am	
Glucagon administered intramuscularly 1 mg (1.5 mg if patient weighs more than 90 kg)	

*Sampling and measurements:*	
Serum GH and capillary blood glucose levels at 0, 30, 60, 90, 120, 150, 180, 210, and 240 mins	

*Normal response:*	
Blood glucose usually rises to peak around 90 mins and then gradually declines (not used to interpret the test)	
Serum GH: peak GH levels tend to occur between 120 to 180 mins with GH levels peaking to above 3 ng/mL	

*Interpretation:*	
In adults with GHD, peak GH levels fail to rise above 3 ng/mL	
